# Cellular uptake and localization of inhaled gold nanoparticles in lungs of mice with chronic obstructive pulmonary disease

**DOI:** 10.1186/1743-8977-10-19

**Published:** 2013-05-16

**Authors:** Marianne Geiser, Oliver Quaile, Alexander Wenk, Christoph Wigge, Sylvie Eigeldinger-Berthou, Stephanie Hirn, Martin Schäffler, Carsten Schleh, Winfried Möller, Marcus A Mall, Wolfgang G Kreyling

**Affiliations:** 1 Institute of Anatomy, Medical Faculty, University of Bern, CH-3012 Bern, Switzerland; 2 Institute of Lung Biology and Disease/Comprehensive Pneumology Center, Helmholtz Center Munich – German Research Center for Environmental Health, D-85764 Neuherberg/Munich, Germany; 3 Department of Translational Pulmonology, Translational Lung Research Center, Member of the German Center for Lung Research, University of Heidelberg, D-69120 Heidelberg, Germany; 4 Division of Pediatric Pulmonology & Allergy and Cystic Fibrosis Center, Department of Pediatrics ІІІ, University of Heidelberg, D-69120 Heidelberg, Germany; 5 Current address: Walter Brendel Centre of Experimental Medicine, Ludwig-Maximilians-University, D-81377 Munich, Germany; 6 Current address: BSL BIOSERVICE Scientific Laboratories GmbH, D-82152 Planegg/Munich, Germany; 7 Current address: Institute of Epidemiology 2, Helmholtz Center Munich – German Research Center for Environmental Health, D-85764 Neuherberg, Munich, Germany

**Keywords:** Aerosol, COPD, Electron microscopy, Gold nanoparticles, Inhalation, Lungs, Macrophages, Stereology

## Abstract

**Background:**

Inhalative nanocarriers for local or systemic therapy are promising. Gold nanoparticles (AuNP) have been widely considered as candidate material. Knowledge about their interaction with the lungs is required, foremost their uptake by surface macrophages and epithelial cells.

Diseased lungs are of specific interest, since these are the main recipients of inhalation therapy. We, therefore, used Scnn1b-transgenic (Tg) mice as a model of chronic obstructive pulmonary disease (COPD) and compared uptake and localization of inhaled AuNP in surface macrophages and lung tissue to wild-type (Wt) mice.

**Methods:**

Scnn1b-Tg and Wt mice inhaled a 21-nm AuNP aerosol for 2 h. Immediately (0 h) or 24 h thereafter, bronchoalveolar lavage (BAL) macrophages and whole lungs were prepared for stereological analysis of AuNP by electron microscopy.

**Results:**

AuNP were mainly found as singlets or small agglomerates of ≤ 100 nm diameter, at the epithelial surface and within lung-surface structures. Macrophages contained also large AuNP agglomerates (> 100 nm). At 0 h after aerosol inhalation, 69.2±4.9% AuNP were luminal, i.e. attached to the epithelial surface and 24.0±5.9% in macrophages in Scnn1b-Tg mice. In Wt mice, 35.3±32.2% AuNP were on the epithelium and 58.3±41.4% in macrophages. The percentage of luminal AuNP decreased from 0 h to 24 h in both groups. At 24 h, 15.5±4.8% AuNP were luminal, 21.4±14.2% within epithelial cells and 63.0±18.9% in macrophages in Scnn1b-Tg mice. In Wt mice, 9.5±5.0% AuNP were luminal, 2.2±1.6% within epithelial cells and 82.8±0.2% in macrophages. BAL-macrophage analysis revealed enhanced AuNP uptake in Wt animals at 0 h and in Scnn1b-Tg mice at 24 h, confirming less efficient macrophage uptake and delayed clearance of AuNP in Scnn1b-Tg mice.

**Conclusions:**

Inhaled AuNP rapidly bound to the alveolar epithelium in both Wt and Scnn1b-Tg mice. Scnn1b-Tg mice showed less efficient AuNP uptake by surface macrophages and concomitant higher particle internalization by alveolar type I epithelial cells compared to Wt mice. This likely promotes AuNP depth translocation in Scnn1b-Tg mice, including enhanced epithelial targeting. These results suggest AuNP nanocarrier delivery as successful strategy for therapeutic targeting of alveolar epithelial cells and macrophages in COPD.

## Background

During the last decade, research on interactions between nanoparticles (NP) and lung tissue has been intensified, especially in terms of potential health risk of particle inhalation. The fast development of nanotechnology has also brought about novel delivery strategies for inhalative and target-specific drugs for therapeutic application, vaccines and diagnostics [1]. There are various definitions of nano-sized materials. On 18 October 2011 the European Commission adopted the recommendation on the definition that “nanomaterial means a natural, incidental or manufactured material containing particles, in an unbound state or as an aggregate or as an agglomerate and where, for 50% or more of the particles in the number size distribution, one or more external dimensions is in the size range 1 nm – 100 nm” [2]. Respiratory tract anatomy, breathing pattern and particle size are the main factors determining particle deposition and retention in the different pulmonary compartments [3]. Since NP deposit with high efficiency in the entire respiratory tract, an increasing number of studies suggest a potential role of inhaled NP to activate and modulate pathophysiological pathways in lungs, which are known to be involved in a range of respiratory diseases, such as chronic obstructive pulmonary disease (COPD), asthma and carcinoma [4]. Gold nanoparticles (AuNP) have also many applications in nanomedicine, as they are apparently non-toxic, suitable for biomedical imaging and can be coated with medical and pharmacological substrates [5].

In lung defense, one of the key players is the surface macrophage. Directly exposed to the environment, it is one of the first cells that comes into contact with inhaled and deposited particles. Thus, its primary function is the phagocytic uptake of deposited particles to keep the lung surface clean, but this also minimizes particle action on the epithelium, uptake by the epithelium or depth translocation beyond the epithelial barrier [6].

Chronic obstructive pulmonary disease (COPD) is one of the main human chronic inflammatory lung diseases and has evolved as the fourth leading cause of death worldwide [7]. Its pathophysiology is caused by inhaled noxious particles and/or gases that trigger inflammatory responses in the larger airways, i.e. chronic bronchitis, as well as a continuous destruction of the alveolar epithelium, i.e. emphysema. Symptomatically, patients suffer from cough and dyspnea resulting from increased mucus secretion and airflow obstruction with air trapping. It was reported that COPD patients have a reduced NP clearance [8] caused by decreased phagocytic activity of alveolar macrophages [9].

Recently a murine model has been developed that recapitulates key features of human COPD [10-12]. The overexpression of the β-epithelial Na^+^-channel (βENaC) encoded by the *Scnn1b* gene causes airway surface dehydration and impaired mucociliary clearance producing chronic mucostasis and airway inflammation. As a result, Scnn1b-transgenic (Tg) animals develop spontaneous chronic bronchitis with goblet cell metaplasia and mucus hypersecretion and emphysema with increased lung volumes, distal airspace enlargement and increased lung compliance [11,13].

The aim of this study was to compare the distribution and localization of AuNP after inhalation in lung tissue, as well as AuNP uptake by surface macrophages of Scnn1b-Tg mice and wild-type (Wt) littermates, using unbiased stereology. To achieve this goal, animals inhaled an aerosol of spark generated AuNP for 2 h. At two time points after inhalation, i.e. immediately (0 h) and at 24 h, surface macrophages harvested by bronchoalveolar lavage (BAL) and whole lungs were prepared for transmission electron microscopic (TEM) analysis by unbiased stereology.

## Results

### AuNP distribution and localization in lung tissue

**Figure 1:**
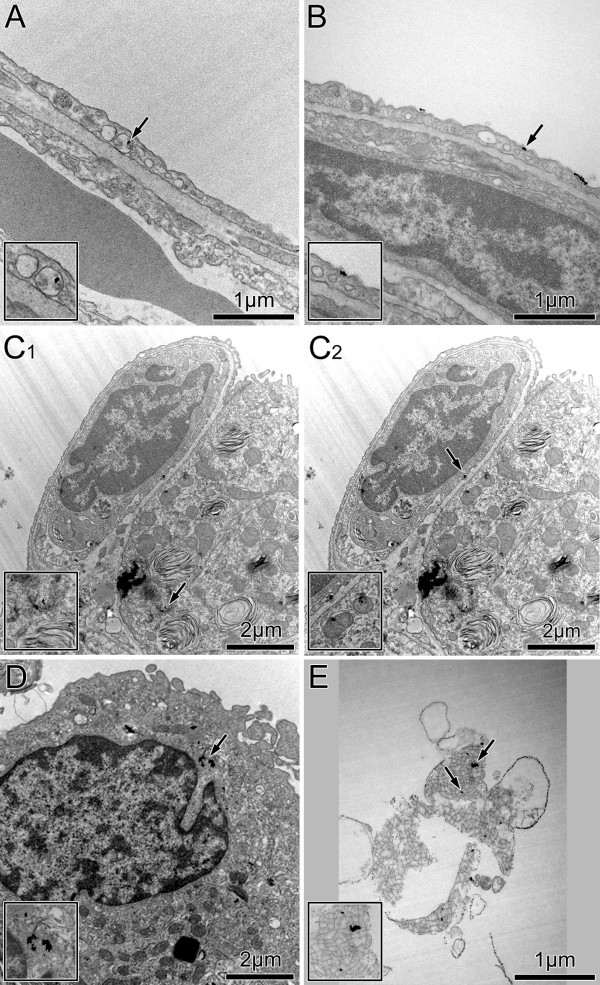
**Representative TEM images of AuNP in lung tissue.** Singlets and small AuNP agglomerates. **A**) Intracellular within a vesicle of an alveolar type I epithelial cell. **B**) Luminal, i.e. attached to the cell membrane of an alveolar type I epithelial cell. **C**_**1**_) In a vesicle of an alveolar type II epithelial cell. **C**_**2**_) In a vesicle of an endothelial cell. **D**) In a vesicle of an alveolar macrophage. **E**) Attached to lining layer material, i.e. surfactant. Arrows point to the regions shown at higher magnification in the inserts.

The well-defined shape and the strong amplitude contrast allow unambiguous identification of AuNP in ultrathin tissue sections by conventional TEM. As demonstrated in Figure 1, AuNP were found in all constituents of the inner lung surface, i.e. in the alveolar lining layer (surfactant), in surface macrophages, attached to the epithelial surface, as well as within alveolar type I and type II epithelial cells. Rarely and upon qualitative examination of lung tissue only, AuNP were also found beyond the epithelial barrier in endothelial cells (Figure 1C_2_). AuNP were not found in the connective tissue. Intracellular AuNP were predominantly localized in vesicles, adjacent to the organelle’s membrane. Very rarely and in epithelial cells only, intracellular AuNP were found in the cytoplasm. AuNP were mainly found as singlets or small agglomerates of ≤ 100 nm diameter. Macrophages also contained large AuNP agglomerates (> 100 nm).

**Figure 2:**
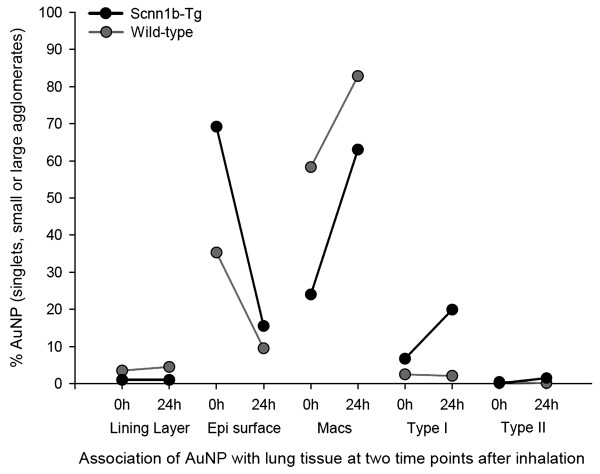
**Relative distribution of AuNP (singlets, small or large agglomerates) in lung tissue.** From 0 h to 24 h after aerosol inhalation, the percentage of lung-retained AuNP decreased on the epithelial surface and increased in macrophages in both animal groups, while in alveolar type I epithelial cells, an increase of AuNP was observed in Scnn1b-Tg mice only. At 0 h, the percentage of AuNP was highest in macrophages of Wt mice, whereas in Scnn1b-Tg mice, the highest percentage of AuNP were registered on the epithelial surface. The percentage of AuNP attached to lining layer material (surfactant) and internalized by alveolar type II epithelial cells was low (less than 5%) in both animal groups and remained unchanged between the two time points studied. Data are presented as mean values of n = 2 animals per genotype and time point.

The stereological analysis of AuNP distribution in lung tissue is shown in Table 1 and Figure 2. The data give evidence for changes in particle distribution between the two time points analyzed, as well as between Scnn1b-Tg and Wt animals: (i) at 0 h after the two-hour aerosol inhalation, about two thirds (69.2 ± 4.9%) of AuNP were found extracellularly on the luminal side, i.e. attached to the epithelial surface, and one quarter (24.0 ± 5.9%) were within macrophages in Scnn1b-Tg mice. In Wt mice, however, one third (35.3 ± 32.2%) of AuNP were attached to the epithelial surface and more than half (58.3 ± 41.4%) were within macrophages. (ii) The percentage of luminal AuNP decreased from 0 h to 24 h after aerosol inhalation, to 15.5 ± 4.8% in Scnn1b-Tg and to 9.5 ± 5.0% in Wt mice. This was in conjunction with an increase of the percentage of intracellular AuNP. (iii) At 24 h, 63.0 ± 18.9% AuNP were within macrophages and 19.9 ± 12.0% within alveolar type I epithelial cells in Scnn1b-Tg mice, while in Wt animals, 82.8 ± 0.2% AuNP were within macrophages and only 2.1 ± 1.5% within alveolar type I epithelial cells. (iv) Less than 5% of AuNP were attached to surface lining layer material (surfactant) or within alveolar type II epithelial cells in both animal groups and at both time points studied.

**Table 1:** Relative distribution of AuNP (singlets, small or large agglomerates) in lung tissue compartments [%]

Animals*	**Lining layer**	**Macs**	**Epithelium**				
			**Epithelial surface**	**Type Icells**	**Type II cells**	**Type I & Type II**	**Total epithelium**
**Tg 0 h**	1.0 ± 1.4	24.0 ± 5.9	69.2 ± 4.9	6.7 ± 1.2	0.1 ± 0.2	6.8 ± 1.0	76.0 ± 5.9
**Tg 24 h**	1.0 ± 1.4	63.0 ± 18.9	15.5 ± 4.8	19.9 ± 12.0	1.5 ± 2.1	21.4 ± 14.2	37.0 ± 19.0
**Tg All**	*1.0 ± 1.2*	*43.5 ± 25.3*	*42.4 ± 31.3*	*13.3 ± 10.4*	*0.8 ± 1.5*	*14.1 ± 11.8*	*56.5 ± 25.3*
**Wt 0 h**	3.5 ± 4.9	58.3 ± 41.4	35.3 ± 32.2	2.5 ± 3.5	0.5 ± 0.7	3.0 ± 4.2	38.3 ± 36.4
**Wt 24 h**	4.5 ± 6.4	82.8 ± 0.2	9.5 ± 5.0	2.1 ± 1.5	0.1 ± 0.1	2.2 ± 1.6	11.7 ± 6.6
**Wt All**	*4.0 ± 4.7*	*70.5 ± 27.8*	*22.4 ± 24.0*	*2.3 ± 2.2*	*0.3 ± 0.5*	*2.6 ± 2.7*	*25.0 ± 26.3*

Tg, Scnn1b-transgenic mice; Wt, wild-type mice. Macs, macrophages, Type I and Type II cells, alveolar epithelial cells. Total epithelium, all AuNP associated with the alveolar epithelium, i.e. attached to and within alveolar (type I and type II) epithelial cells.

Data are presented as the mean ± SD.

*, n = 2 per genotype and time point.

As shown in Table 2, there was no significant statistical evidence of any mean fine pulmonary structure difference between Scnn1b-Tg and Wt mice. In addition, we found no evidence for any significant recruitment of macrophages to the lung surface after aerosol inhalation, as macrophage numbers were not different in lungs fixed at 0 h and 24 h after inhalation of the aerosol in any experimental group. As expected, lung volumes [14] were generally larger in Scnn1b-Tg (range: 1.41 - 1.56 mL, n = 4) than in Wt mice (range: 1.08 - 1.46 mL, n = 4) [11,13], however, without reaching statistical significance for the number of mice included in this study.

**Table 2:** Lung volume and fine pulmonary structure

**Animals**	**V(lung) [ml]**	**Air/tissue [%]**	**Tissue distribution [%]**				
			**Connective tissue**	**Capillaries**	**Macs 0 h/24 h**	**Type I cells**	**Type II cells**
**Tg (n = 4)**	1.48 ± 0.06	86.0 ± 0.8/14.0 ± 0.8	36.8 ± 14.2	38.2 ± 19.6	2.9 ± 2.6/2.0 ± 2.2	19.1 ± 6.9	3.5 ± 0.4
**Wt (n = 4)**	1.27 ± 0.21	86.0±0.6/13.5±1.4	32.4 ± 11.0	36.3 ± 24.4	3.1 ± 3.0/3.9 ± 4.7	23.3 ± 11.0	4.9 ± 3.3

V (lung), lung volume [14]; Tg, Scnn1b-transgenic mice; Wt, wild-type mice; Macs, macrophages. Type I and Type II cells, alveolar epithelial cells. Data are presented as the mean ± SD.

### AuNP uptake in BAL macrophages

**Figure 3:**
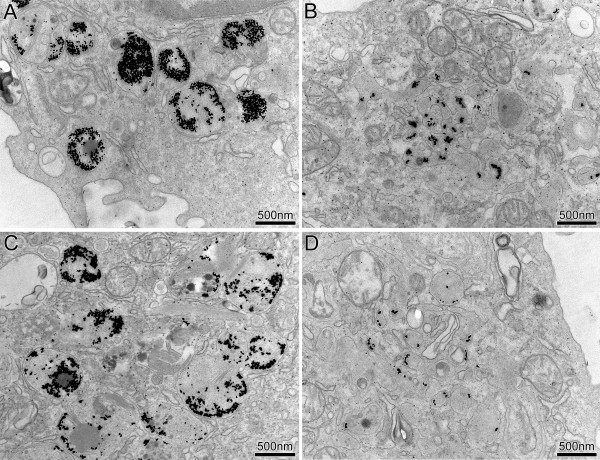
**Representative TEM images of AuNP in BAL macrophages. A**, **B**) Scnn1b-Tg mice. **C**, **D**) Wt mice. **A**, **C**) Large AuNP agglomerates (> 100 nm in diameter) were predominantly localized in medium-sized (150 – 1000 nm) and large vesicles (> 1000 nm). **B**, **D**) Single or small AuNP agglomerates (< 100 nm) were localized in small vesicles (< 150 nm), whereby the vesicle diameters still largely exceeded particle size. Note the close localization of the AuNP to the organell’s membrane.

In BAL macrophages, AuNP were found as single particles and agglomerates of variable sizes, exclusively in vesicles. For a more detailed analysis of AuNP uptake by macrophages, vesicles containing AuNP were subdivided into the following size categories: (i) small primary vesicles (< 150 nm in diameter), (ii) medium-sized vesicles (150 – 1000 nm) such as endosomes or multi-vesicular bodies and (iii) large vesicles (> 1000 nm) such as phagosomes or lysosomes [6]. As depicted in Figure 3, single AuNP or small agglomerates were found in small vesicles, whereby vesicle size still largely exceeded particle size. AuNP agglomerates of > 100 nm in diameter were predominantly localized in the medium-sized and large vesicles, which usually contained no other electron-dense material. Association of AuNP with the vesicular membrane was observed for particles in vesicles of all size categories.

The data of AuNP uptake in BAL macrophages, assessed by the relative deposition index (RDI) [15,16], are shown in Table 3. The results demonstrate that the number of observed particles in macrophages (*N*_*O*_) is significantly higher (RDI > 1) than the expected number of particles (*N*_*E*_) in Wt animals at 0 h (RDI = 1.3) and in Scnn1b-Tg mice at 24 h (RDI = 1.2) after aerosol inhalation. For the remaining two animal groups, the number of observed particles in macrophages was lower than expected (RDI < 1), i.e. not significantly different from what could be expected.

**Table 3:** Uptake of AuNP in BAL macrophages at 0 h and 24 h after aerosol inhalation

**Animals**	**N**_**O**_	**N**_**P**_	**N**_**E**_	**RDI**	**Partial χ**^**2**^	**Partial χ**^**2**^**as%**
**Wt 0 h**	95	35392	75.7	1.3*	4.9	37.6
**Wt 24 h**	52	33328	71.3	0.7	5.2	62.4
***Total***	*147*	*68720*			*10.1*	
**Tg 0 h**	29	18688	42.2	0.7	4.1	48.5
**Tg 24 h**	83	30944	69.8	1.2*	2.5	51.7
***Total***	*112*	*49632*			*6.6*	

N_O_, number of observed particles; N_P_, number of observed points (hitting a macrophage) per animal; N_E_, number of expected particles; RDI, relative deposition index. *RDI > 1 and partial χ^2^ > 30% indicate a preferentially populated compartment, i.e. more AuNP were found than expected for random AuNP distribution. Tg, Scnn1b-transgenic mice; Wt, wild-type mice.

## Discussion

Unbiased stereology combined with TEM allows quantitative assessment of the ultrastructural localization and distribution of inhaled NP in lungs at the individual particle level. In the present study, we investigated the distribution of inhaled 21-nm AuNP in whole lungs and in BAL macrophages in a transgenic mouse model of COPD in comparison to wild-type mice, at 0 h and 24 h after the inhalation of the aerosol by negative-pressure ventilation for 2 h.

Stereological analysis of particle distribution in lungs demonstrated a rapid association of AuNP with the epithelial surface, i.e. alveolar type I epithelial cells, which cover 95% or more of the alveolar surface area [17]. These data are in agreement with results of a recent study about the influence of surfactant protein D on the biodistribution of inhaled 22-nm AuNP in mice, where a high fraction of AuNP retained in lungs were shown to be associated with the lung tissue [18]. Ultrastructural TEM analysis allowed in addition distinguishing AuNP attached to the cell membrane from those internalized. The results revealed similarities but also differences between the two animal groups, as well as between the two time points studied: Overall, the percentage of AuNP associated with the alveolar epithelium, i.e. those attached to and within alveolar type I or type II epithelial cells (Table 1) was substantially higher in Scnn1b-Tg (56.5 ± 25.3%, mean over all animals and time points) than in Wt lungs (25.0 ± 26.3%). Conversely, the percentage of AuNP was substantially higher in macrophages of Wt (70.5 ± 27.8%, mean values over all animals and time points) than of Scnn1b-Tg animals (43.5 ± 25.3%). In both animal groups, the percentage of AuNP attached to the epithelium was lower at 24 h than at 0 h and concomitantly, the percentage of AuNP in macrophages was higher at 24 h than at 0 h after aerosol inhalation. In Wt animals, AuNP appeared to be rapidly removed from the alveolar surface by macrophages. In Scnn1b-Tg mice, however, uptake of AuNP by surface macrophages appeared to be less efficient. In addition, a substantial part of AuNP was found internalized by the primary surface-covering alveolar epithelial type I cells in Scnn1b-Tg mice; 6.7 ± 1.2% at 0 h and 19.9 ± 12.0% at 24 h after the inhalation of the aerosol. Less efficient uptake of AuNP by surface macrophages in lungs of Scnn1b-Tg mice was confirmed by the results of AuNP internalization in BAL macrophages, which is further described below. Hence, the results from lung tissue analysis provide evidence for a prolonged residence time of AuNP on the lung surface in chronic obstructive lung disease in Scnn1b-Tg mice resulting in increased NP uptake by epithelial cells. These findings are of interest for therapeutic targeting of epithelial cells in COPD/emphysema. A prolonged residence time of AuNP on the alveolar surface and augmented AuNP uptake by the primary surface-covering alveolar type I epithelial cells in addition enhance the probability of AuNP translocation into the adjacent endothelial cells and into the blood capillaries and, hence, further systemic distribution of AuNP [19]. In case of inhaled harmful NP, this may be an important factor in disease initiation or for alterations in the course of disease, i.e. exacerbation.

Because stereological analysis did not result in any AuNP in the connective tissue, we consider particle accumulation in this lung compartment to be negligible. However, we have evidence for AuNP translocation beyond the epithelium from qualitative TEM analysis, as we found AuNP –though a few only– in endothelial cells of capillaries (Figure 1C_2_). Thus, these results are also in agreement with the findings in the AuNP-biodistribution study in mice, where rapid systemic translocation of a small fraction of AuNP was reported [18]. Similarly, in rat lungs, only a small fraction of inhaled AuNP was reported to translocate to the systemic circulation by crossing the air-blood barrier [20,21].

Particle uptake by resident surface macrophages and their further migration to airways and mucociliary transport to the larynx is the main mechanism for particle clearance from the peripheral lungs [19]. In the present study, there is consistent evidence for less efficient AuNP uptake by macrophages in Scnn1b-Tg compared to Wt animals and, hence, for limited macrophage clearance of NP in diseased lungs: (i) data of AuNP distribution in whole lungs (Table 1, Figure 2) revealed considerably lower percentages of AuNP internalized by macrophages in Scnn1b-Tg than in Wt mice at both time points studied (0 h and 24 h); (ii) analysis of AuNP uptake in BAL macrophages (Table 3) revealed a RDI = 1.3 in Wt animals at 0 h, indicating that deposited AuNP were rapidly and more eagerly internalized than at 24 h by the same condition group (RDI = 0.7). In contrast, in Scnn1b-Tg animals, the RDI was 1.2 at 24 h and higher than at 0 h (RDI = 0.7), indicating delayed AuNP clearance from the epithelial surface by macrophages in this animal group.

AuNP in macrophages and in alveolar type I epithelial cells were mainly found in vesicles (Figure 1 and Figure 3). This is in agreement with studies in rats, where AuNP were also observed in vesicles of the same cell types [20,21]. Likewise, in an in vitro study with BAL macrophages, AuNP were found in pinocytic vesicles and lysosomes [22]. This is in line with other reports on intra-vesicular particle localization, suggesting NP uptake by cells to mainly occur by endocytic processes [16]. The variety of vesicular structures in which we observed AuNP indicate different endocytosis mechanisms [23]. AuNP found as singlets or small agglomerates (< 100 nm in diameter) in vesicles of all cell types reflect pinocytic uptake. Vesicles containing large AuNP agglomerates of 500 nm diameters or more, found in macrophages only, demonstrate phagocytosis that allows uptake of micrometer sized objects.

We very rarely found AuNP in the cytoplasm of alveolar type I epithelial cells, suggesting that passive uptake of NP, i.e. not triggered by receptor-ligand interactions, is not a major pathway for AuNP uptake by alveolar epithelial cells. Cytoplasmic localization of inhaled NP has been reported for inhaled 20-nm TiO_2_NP in rat lungs [24]. The difference between TiO_2_ and AuNP in regard to cytoplasmic localization might be explained by the finding that in contrast to AuNP, we did not observe TiO_2_NP bound to the apical surface of epithelial cells in rat lungs. So, it might be that AuNP bind to surface receptors that are constitutively or abundantly expressed on alveolar epithelial cells, while TiO_2_NP do not bind to such receptors but are more likely to enter the cell by non-adhesive forces as we have previously suggested [24].

AuNP attached to the epithelial surface were exclusively found as singlets or small agglomerates. Since we registered macrophages containing large particle agglomerates at the same time points, this may indicate efficient macrophage clearance of agglomerated but not of single AuNP from the lung surface. This is in line with the results of all analyses in this study.

The concomitant finding of single or small agglomerates of AuNP (on the alveolar surface and within any cell types) and of large agglomerates (in macrophages only) is not easily explained. Under the laminar flow conditions in the alveolar region, the deposition mechanism of the 21-nm AuNP is maintained by diffusion leading to a rather homogeneous deposition density throughout the entire alveolar epithelium. Estimating the cross sectional surface of all deposited AuNP in relation to the alveolar surface area of 400 cm^2^ in mice (see Table 4 and Table 5), the statistical mean distance between two deposited AuNP is about half of the mean AuNP diameter of 21 nm. Hence, coagulation of neighboring AuNP can only occur to a limited extent leading to AuNP singlets and small agglomerates. This is in agreement with our findings of AuNP singlets and small agglomerates on and within alveolar epithelial cells, within endothelial cells and within some macrophages. Following our estimate on the AuNP density on the epithelial surface, the large agglomerates, which were exclusively observed in macrophages, may be indicative for intracellular fusion of vesicles and, hence, a “retrograde” formation of larger agglomerates inside macrophages. Such large AuNP agglomerates were also found in a previous inhalation study in rats using similar AuNP types, however, preferentially at later time points [20,21].

**Table 4:** Characteristics of the inhaled AuNP aerosol

**Parameter**	**Value**
**Aerosol concentration (p/cm**^**3**^**),** continuously measured and averaged over the entire study time; subsequent averaging of data over all inhalation periods	1.28 ± 0.08 × 10^7^
**Inhalation period (min),** experimentally set	120
**Tidal volume (cm**^**3**^**),** estimated from pneumotachograph signals	0.18
**Breathing frequency (1/min),** experimentally set	120
**Minute volume (cm**^**3**^**/min),** calculated from data above	21.6
**Inhaled volume (L/120 min),** calculated from data above	2.6
**Deposition fraction,** based on MPPD model	0.4
**CMD of AuNP (nm)/GSD,** measured continuously as described in text and averaged over all inhalation periods	21/1.6
**Number of AuNP inhaled,** calculated from data above	3.3 × 10^10^
**Number of AuNP deposited in lungs,** calculated from data above	1.3 × 10^10^

MPPD, multiple-path particle dosimetry (computational model to estimate human and rodent particle dosimetry). CMD, count median diameter; GSD, geometric standard deviation.

**Table 5:** Calculated AuNP deposition density on the alveolar epithelium

**Parameter**	**Value**
**CMD of Au NP (nm)**	21
**Volume of AuNP (cm**^**3**^**),** calculated median volume of spherical AuNP with density given below	4.9 × 10^-18^
**Nominal density of Au (g/cm**^**3**^**)**	19.3
**Mass of AuNP (g),** calculated from data above	9.4 × 10^-17^
**AuNP aerosol mass concentration (mg/m**^**3**^**),** calculated from data in Table 4 and Table 5	1.20
**Number of AuNP deposited in lungs, 120 min,** calculated from data in Table 4 and Table 5	1.3 × 10^10^
**AuNP deposited mass, 120 min (g)** calculated from data in Table 4 and Table 5	1.24 × 10^-6^
**Total projected area of all AuNP deposited (cm**^**2**^**)** calculated from data above	2.2 × 10^-10^
**Lung surface area (cm**^**2**^**),**[25]	400
**Fraction of NP coverage of lung surface (cm**^**2**^**)** calculated from data above	5.5 × 10^-13^
**Mean linear distance between 2 NP (nm),**[25]	7.4

CMD, count median diameter.

## Conclusions

This, to our knowledge, first study on the ultrastructural distribution of inhaled AuNP by unbiased stereology in Scnn1b-Tg mice with COPD and healthy Wt littermates gives further insight into the interaction of deposited NP with fine pulmonary structures. We observed rapid binding of AuNP to the alveolar epithelium, i.e. to the primary surface-covering alveolar type I epithelial cells in both animal groups. In Scnn1b-Tg mice, we found delayed and less uptake of AuNP by surface macrophages and concomitant higher AuNP uptake by alveolar type I epithelial cells compared to Wt mice. In case of inhaled harmful NP, this may be an important factor in disease initiation or for alterations in the course of disease, i.e. exacerbation. Conversely, a prolonged residence time of deposited AuNP on the epithelial surface and increased NP uptake by the alveolar epithelium may be favorable for therapeutic targeting of the lung parenchyma by inhaled aerosols in COPD/emphysema. To unravel NP-lung interaction, more studies with diseased lungs are required.

## Materials and methods

### Experimental design

To resolve the interaction of AuNP with the inner lung surface, we studied (i) AuNP distribution in lung tissue and (ii) AuNP uptake by surface macrophages, at the individual particle level, immediately (0 h) and 24 h after a 2-hour aerosol inhalation, in Scnn1b-Tg mice and Wt littermates.

### Animals

Scnn1b-Tg mice (congenic line generated in a mixed C3H/HeN:C57BL/6 N background [11]) and Wt littermates were bred and reared at the University of Heidelberg, Heidelberg, Germany. Mouse progeny were genotyped by tail biopsy polymerase chain reaction (PCR) as previously described [26]. Specified pathogen-free status was approved by a health certificate according to Federation of European Laboratory Animals Science Association (FELASA) guidelines. For the experiments, mice were transferred to the animal facility of the Institute of Lung Biology and Disease at the Helmholtz-Center Munich, Neuherberg/Munich, Germany. They were housed in individually ventilated cages (IVC; BioZone, Ramsgate, UK) supplied with filtered air and access to food and water ad libitum. Mice were kept on a 12 h day/night cycle, humidity was maintained at 55% and the temperature was 22°C.

All experiments were conducted under federal guidelines for the use and care of laboratory animals and were approved by the Bavarian Animal Research Authority and by the Helmholtz Center’s Institutional Animal Care and Use Committee, the Animal Care and Use Committee of the Regierungspräsidium Karlsruhe, Germany, as well as in accordance with the Swiss Federal Act on Animal Protection and the Swiss Animals Protection Ordinance.

Six Scnn1b-Tg and six Wt mice (females, age: 10 – 18 weeks, body weights: 22 - 33 g) were used. There were no significant differences in age or body weight between mutant and Wt mice. For particle inhalation and for lung fixation prior to euthanasia by exsanguination via the abdominal aorta, animals were anesthetized by intraperitoneal (i.p.) injection of a mixture of medetomidine (Domitor®, Pfizer GmbH, Karlsruhe, Germany; 0.5 mg/kg body weight), midazolam (Dormicum®, Hoffmann-La Roche AG, Grenzach-Wyhlen, Germany; 5 mg/kg) and fentanyl (Fentanyl®, Janssen-Cilag GmbH, Neuss, Germany; 0.05 mg/kg). For examinations at 24 h, anesthesia was antagonized by subcutaneous injection of atipamezole (Antisedan®, Pfizer GmbH Karlsruhe, Germany; 2.5 mg/kg), flumazenil (Anexate®, Hoffmann-La Roche AG, Grenzach-Wyhlen, Germany; 0.5 mg/kg), and naloxone (Narcanti®, Janssen Animal Health, Neuss, Germany; 1.2 mg/kg).

### Aerosol generation and inhalation

**Figure 4:**
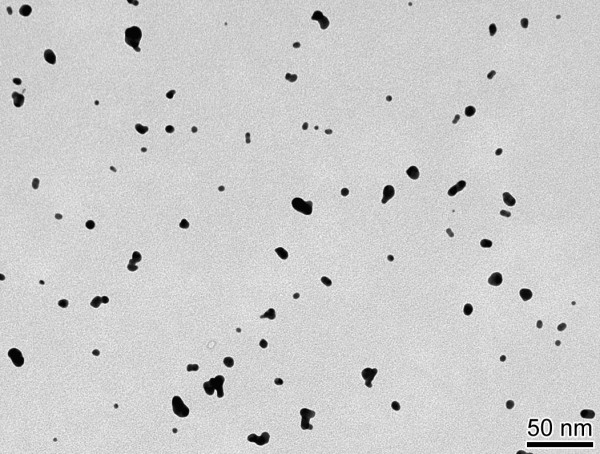
**Representative TEM image of AuNP aerosol.** Morphology of freshly generated AuNP aerosol for inhalation collected on formvar-coated copper grids using a TEM particle sampler (University of Applied Sciences). The spherical shape was obtained after melting of AuNP in the airborne state by heat treatment at 600°C [28].

AuNP aerosol generation and inhalation were performed as previously described for titanium dioxide NP [27] and gold NP [28]; relevant data are shown in Table 4 and Table 5. Briefly, AuNP aerosols were generated with a spark generator (GFG100, Palas, Karlsruhe, Germany), quasi-neutralized with a radioactive ^85^Kr source, heat-treated at 600°C in a tube furnace for melting the AuNP agglomerates to spherical AuNP, diluted and conditioned for inhalation in terms of gas composition, humidity and temperature. Particle number concentration and size distribution were continuously monitored by a condensation particle counter (CPC 3022A, TSI, Aachen, Germany) and a scanning differential electrical mobility particle sizer (SMPS, Classifier 3071 and CPC 3010, TSI), respectively. The aerosol produced for inhalation had a count median diameter (CMD) of about 21 nm (geometric standard deviation, GSD = 1.6) and a mean number concentration of 1.28 ± 0.08 × 10^7^ nanoparticles/cm^3^ throughout the exposures; due to the high gold density of 19.3 g/cm^3^ the estimated mass concentration was 1.2 mg/m^3^. In addition, AuNP were sampled for morphologic analysis on filters and on formvar-coated copper grids after aerosol generation using a TEM particle sampler (University of Applied Sciences, Windisch, Switzerland). A representative image of the inhaled aerosol is shown in Figure 4. Anesthetized mice were placed in airtight plethysmograph boxes for 2 h and inhaled the aerosol via an endotracheal tube by negative-pressure ventilation (−1.5 kPa) with 0.25 sec of inspiration followed by 0.25 sec of expiration at ambient air pressure, resulting in a breathing frequency of 120 breaths/min. The deposited amount of AuNP was calculated to be 1.24 μg or 1.3 × 10^10^ NP in each mouse lung assuming a 40% deposition probability. We used the CMD to estimate the deposition fraction, since under the given thermodynamic conditions during breathing a mass median aerosol diameter (MMAD) is not defined due to negligible sedimentation and deposition is only determined by diffusion, which is independent of the density of AuNP of 21 nm diameter. During the second hour of ventilation, 1.5% isoflurane gas was added to the aerosol in order to maintain the mice under anesthesia.

### Lung processing and stereology

#### Lung tissue

Lungs (n = 8, two per genotype and time point) were fixed in situ by airway instillation of phosphate-buffered 2.5% glutaraldehyde (Agar Scientific Ltd., Stansted, Essex, England; 350 mOsm, pH 7.4) at a pressure of 25 cm H_2_O, as previously described [29]. Thereafter, lungs were removed *in toto* from the thorax and stored for at least 24 h in fixative solution at 4°C. Lungs were then subjected to systematic tissue sampling, post-fixed with buffered 1.0% osmium tetroxide (Simec, Zofingen, Switzerland) and 0.5% uranyl acetate (Fluka Chemie GmbH, Sigma-Aldrich, Buchs, Switzerland), dehydrated in a graded series of ethanol and acetone, and finally embedded in epon (Fluka), as previously described [19,30]. Ultrathin sections (≤ 50 nm) were cut from 6 - 8 tissue blocks per animal, mounted onto formvar-coated 200-mesh copper grids and post-stained with uranyl acetate and lead citrate (Ultrastain; Leica, Glattbrugg, Switzerland). Ultrathin lung sections, i.e. twelve systematic fields (every second field with random start) per section, were analyzed for (i) lung tissue composition at 400 × magnification and (ii) AuNP morphology and localization at 7100 × magnification, using a FEI Morgagni 268D TEM operated at 80 kV. AuNP were analyzed according to their shape and size (single or agglomerated) as well as in regard to their anatomical-histological and sub-cellular localization. The following associations with the lung tissue were discerned: surface macrophage, alveolar type I and type II epithelial cells, alveolar lining layer, luminal, i.e. attached to the epithelial surface, and connective tissue.

#### BAL macrophages

To recover surface macrophages, lungs (n = 4, one animal per genotype and time point) were lavaged in situ with 5 × 1 ml divalent cation free phosphate buffered saline (PBS) (Sigma-Aldrich, Taufkirchen, Germany) under gentle massage of the thorax [31]. To prevent further particle uptake by cells, recovered BAL fluid was immediately mixed with equal amounts of phosphate-buffered 2.5% glutaraldehyde. Thereafter, BAL fluid was centrifuged, cells were resuspended in fresh glutaraldehyde and then further prepared for TEM analysis as described above. Ultrathin sections of the cell pellets were stereologically analyzed for AuNP uptake by surface macrophages with a CM12 TEM operated at 80 kV (Philips, Eindhoven, The Netherlands).

#### Stereology and statistics

Stereological analysis of TEM images from the systematic fields sampled on ultrathin tissue sections was performed with the STEPanizer software version 1 [15,32]. The fine pulmonary structure and AuNP uptake in BAL macrophages were evaluated using point counting test systems. Data of Scnn1b-Tg and Wt animals were compared using the nonparametric Mann–Whitney Rank Sum Test. The level of significance was set at p < 0.05.

AuNP distribution within BAL macrophages was assessed by the relative deposition index (RDI), which indicates whether the number of observed particles in a defined compartment (*N*_*O*_) is higher (RDI > 1), equal (RDI = 1) or lower (RDI < 1) than the expected number of particles (*N*_*E*_) that could be predicted considering the compartment volume [15,16]. If the observed number of particles, *N*_*O*_, equalizes or is below the expected number of particles, *N*_*E*_, the distribution of NP follows a random distribution among the compartment studied. Here the compartment of interest was the BAL macrophage, in which AuNP distribution was compared between Scnn1b-Tg and Wt animals. The expected number of particles for each animal, *N*_*E*_, was calculated from the total number of observed AuNP, *N*_*O*_*(total)*, the number of points observed within the compartment of interest at one time point (0 h or 24 h), *N*_*P*_ and the total number of observed points within the compartment of interest (*N*_*P*_*(total)*) using the following equation:

$$N_{E}=N_{O}\;\left(\mathrm{total}\right)\;\times \;\left(N_{P}/N_{P}\left(\mathrm{total}\right)\right)$$

With *N*_*O*_ and *N*_*E*_, the relative deposition index (RDI) was calculated for each animal:

$$\mathrm{RDI}=N_{O}/N_{E}$$

*N*_*O*_ and *N*_*E*_ of Scnn1b-Tg and Wt mice at 0 h and 24 h were then statistically compared using the chi squared(χ^2^) test [15,33]. RDI >  1 and partial *χ*^2^ > 30*%* (*Partial χ*^2^ = (*N*_*O*_ − *N*_*E*_)^2^/*N*_*E*_) indicate a preferentially particle loaded compartment, meaning here that a higher number of AuNP were found within BAL macrophages than expected for random AuNP distribution.

## Abbreviations

Au: Gold; BAL: Bronchoalveolar lavage; βENaC Scnn1b: β subunit of epithelial Na^+^ channel encoded by the Scnn1b gene; CMD: Count median diameter; COPD: Chronic obstructive pulmonary disease; CPC: Condensation particle counter; i.p.: Intraperitoneal; NP: Nanoparticles; PBS: Phosphate buffered saline; TEM: Transmission electron microscopy; SMPS: Scanning differential electrical mobility particle sizer; GSD: Geometric standard deviation; Tg: Transgenic; Wt: Wild type.

## Competing interests

The authors declare that they have no competing interests related to this manuscript.

## Authors’ contributions

The authors responsibilities were as follows – MG, WGK, WM, CS and MAM: designed the research; MAM generated the animal model. MG, WGK, AW, SH, MS, CS, OQ and CW conducted the research. MG, OQ, WGK and SEB wrote the manuscript and had primary responsibility for final content. All authors critically read and approved the final manuscript.
